# Polyphenol-rich extract from blackcurrant pomace attenuates the intestinal tract and serum lipid changes induced by a high-fat diet in rabbits

**DOI:** 10.1007/s00394-014-0665-4

**Published:** 2014-02-06

**Authors:** Adam Jurgoński, Jerzy Juśkiewicz, Zenon Zduńczyk, Paulius Matusevicius, Krzysztof Kołodziejczyk

**Affiliations:** 1 Division of Food Science, Institute of Animal Reproduction and Food Research, Polish Academy of Sciences, 10 Tuwima Street, 10-748 Olsztyn, Poland; 2 Lithuanian Veterinary Academy, Lithuanian University of Health Sciences, 9 Mickevičiaus Street, 44307 Kaunas, Lithuania; 3 Institute of Chemical Technology of Food, Technical University of Łódź, 4/10 Stefanowskiego Street, 90-924 Łódź, Poland

**Keywords:** Blackcurrant, Polyphenols, Pomace, Serum lipids, Antioxidant status, Caecum

## Abstract

**Purpose:**

The consumption of a high level of dietary extract from blackcurrant pomace rich in polyphenols was hypothesised to exert beneficial effects on the serum lipid profile, the markers of insulin resistance and the antioxidant status of the host without negative changes in the intestinal tract.

**Methods:**

This hypothesis was tested on 20 male New Zealand white rabbits randomly assigned to four groups of five individuals each. For 4 weeks, the animals were subjected to the following dietary treatments: two control groups were fed a standard or a high-fat diet (7 and 32 % energy from fat, respectively), and two experimental groups were fed a standard or a high-fat diet with the addition of 1.5 % blackcurrant polyphenolic extract. The extract obtained from blackcurrant fruit pomaces was characterised by high concentrations of anthocyanins and flavonols (48.9 and 17.9 %, respectively).

**Results:**

The high-fat feeding regimen led to a series of unfavourable changes, such as increased body weight, disturbance of fermentative processes in the hindgut as well the induction of oxidative stress, hyperlipidaemia and insulin resistance. Dietary supplementation with blackcurrant extract decreased the concentration of putrefactive metabolites (ammonia and putrefactive SCFA) and β-glucuronidase activity in the hindgut digesta. Additionally, the extract ameliorated hyperlipidaemia by decreasing triglyceride, total cholesterol, non-HDL cholesterol and free fatty acid concentrations in the serum and increased the antioxidant capacity of the serum.

**Conclusion:**

This study suggests that a polyphenol-rich extract from blackcurrant pomace ingested at relatively high amounts may be a useful therapeutic option in the reversal of dysfunctions related to obesity and its complications.

## Introduction

Berries are the most widely consumed fruit in human diets. Blackcurrant (*Ribes nigrum* L. Grossulariaceae) berries are known to be rich in polyphenolic compounds (on average 250 mg/100 g of fresh fruit), which have been demonstrated to be potent antioxidants and cardioprotective agents [1]. Blackcurrant fruits and leaves have also been used in traditional medicine to treat a variety of ailments [2]. The consumption of fresh fruit is limited, but blackcurrants are processed commercially in jellies, jams, juices and alcoholic drinks [3]. However, the content of polyphenolic compounds can be reduced during juice processing from the enzymatic depectinisation and pasteurisation processes while simultaneously leaving high amounts of these compounds in the by-products, e.g. pomace [4]. Additionally, the dietary level of polyphenols at which health-promoting effects might be expected, especially in an organism with serious metabolic disturbances, is difficult to obtain through the consumption of fruit and common commercial products. Therefore, one way to utilise the by-products is to produce phenolic extracts [4]. Several studies have indicated the possibility of using extracts from blackcurrant to beneficially modulate the key markers of the health status of a consumers’ body [1, 3, 5].

Clinical and experimental studies have suggested that the proportion of dietary polyphenols absorbed in the small intestine is relatively low (10–20 % depending on the polyphenol chemical structure and origin), and most ingested polyphenols reach the large intestine where they encounter microbiota, which have enormous catalytic and hydrolytic potentials [6, 7]. Some authors have identified adverse effects associated with high dosages of dietary polyphenols in the gastrointestinal environment, including caecal and colonic fermentation processes [8], but well-documented experiments addressing the in vivo effects of dietary blackcurrant polyphenols are scarce [3]. Molan et al. [3] found that rats gavaged with 13.4 mg blackcurrant extract per kg of body weight three times weekly for four consecutive weeks exhibited significantly reduced activity of caecal β-glucuronidase; interestingly, the decrease in β-glucuronidase activity observed in this study was greater than the decrease observed after administration of prebiotic inulin. In an in vitro experiment, Werlein et al. [5] demonstrate that blackcurrant concentrate, which contains different classes of polyphenols, inhibits the growth of *Staphylococcus aureus* DSM 799 and *Enterococcus faecium* DSM 2918, while a purified anthocyanin mix did not influence the growth of these microorganisms. This is supported by Puupponen-Pimiä et al. [9] who claim that the antimicrobial activity of berry extracts results from the synergy of various phenolic compounds.

The present study hypothesises that a high level of consumption of polyphenol-rich extract from blackcurrant pomace produces improvements in the serum lipid profile, the markers of insulin resistance and the antioxidant status of the host without negatively affecting the intestinal tract. To assess these issues, two types of rabbit diets were created (one with standard fat content and another with fat content increased through the addition of lard); one group of rabbits for each diet was supplemented with a blackcurrant polyphenolic extract rich in anthocyanins and flavonols. A high-fat diet seems to be strongly associated with phylogenic changes in the composition of the gut microbiota of obese individuals, likely due to the overflow of dietary fat into the distal intestine [10, 11]. Thus, the addition of lard to the diet was intended to induce adverse effects not only in the serum lipids but also in the hindgut function of the rabbits.

## Materials and methods

### Blackcurrant extract and its chemical analyses

The extract was obtained from blackcurrant fruit pomace, a by-product of the manufacture of concentrated blackcurrant juice (ALPEX Co., Leczeszyce, Poland). Briefly, the fresh pomace from a Bücher type press was dried in a convection oven at a temperature ≤70 °C until the moisture content fell below 5 % and then passed through 2-mm mesh sieves. The seedless preparation thus obtained was then subjected to sequential water and ethanol extraction procedures as described elsewhere in the literature [4]. The final product was a raw blackcurrant extract that was freeze-dried and then utilised as a dietary supplement.

The chemical composition of blackcurrant extract is detailed in Table 1. The extract was analysed in duplicate for dry matter (DM), crude protein (CP), fat, ash and total dietary fibre (TDF) using AOAC methods 934.01, 920.152, 930.09, 940.26 and 985.29, respectively [12]. Nitrogen-free extract (NFE) was calculated by adding CP, fat, water, ash and fibre and subtracting that sum from 100. A high-performance liquid chromatography (HPLC) analysis of the phenolic compounds in the blackcurrant extract was also performed. The polyphenol compounds were extracted utilising a solution composed of methanol/water/formic acid at a volumetric ratio of 50:48:2 v/v/v and identified through a previously described procedure [13]. Before conducting the analysis, the extracts were centrifuged at 4,800×*g* for 5 min. The anthocyanins and other phenolics were analysed using KNAUER Smartline chromatograph (Berlin, Germany) equipped with two pumps. The compounds in the phenolic extracts were separated on a 150 mm × 4.6 mm inner diameter, 5 μm Gemini 5u C18 110A column (Phenomenex Synergi, Torrance, CA, USA) using gradient elution with 10 % v/v formic acid in water (A) and 50:40:10 v/v/v acetonitrile/water/formic acid (B). The column temperature was set to 40 °C. The flow rate was 1 mL/min, and the gradient program was as follows: 0–0.6 min, 12 % B; 0.6–16 min, 12–30 % B; 16–20.5 min, 30–100 % B; 20.5–22 min, 100 % B; 22–25 min, 100–12 % B, 25–35 min, 12 % B. The injection volume was 20 μL. The data were collected by the EuroChrom 2000 program (Knauer GmbH, Berlin, Germany). Quercetin and myricetin glycosides, and their aglycones, were detected at a wavelength of 360 nm, while the anthocyanins were assayed at 520 nm. Standards of cyanidin-3-rutinoside, myricetin and kaempferol-3-glucoside were purchased from Extrasynthese Company (Genay, France); quercetin, kaempferol, quercetin-3-rutinoside and (-) epicatechin were purchased from Sigma-Aldrich (Poznań, Poland). To identify the anthocyanins and the remaining flavonoids, standards were employed and the UV–vis spectra and procedures documented in the literature were employed [13].

**Table 1:** Chemical composition of the blackcurrant extract^a^

	g/100 g of the extract
Dry matter	96.6 ± 0.0
Crude protein	3.6 ± 0.1
Ether extract	0.0 ± 0.0
Crude ash	2.6 ± 0.0
Total dietary fibre (TDF)	10.1 ± 0.3
Nitrogen-free extract (NFE)	13.5 ± 0.2
Total polyphenolic compounds (HPLC–DAD):	66.8 ± 1.9
Total anthocyanins, including:	48.9 ± 1.2
Delphinidin-3-rutinoside^b^	16.6 ± 0.3
Delphinidin-3-glucoside^b^	14.0 ± 0.2
Cyanidin-3-rutinoside^b^	12.4 ± 0.2
Cyanidin-3-glucoside^b^	5.5 ± 0.1
Other anthocyanins	0.4 ± 0.3
Total flavonol aglycones, including:	13.3 ± 0.3
Myricetin	8.3 ± 0.2
Quercetin	3.6 ± 0.1
Kaempferol	1.1 ± 0.0
Isorhamnetin^c^	0.3 ± 0.0
Total flavonol glycosides, including:	4.6 ± 0.1
Myricetin glycosides^d^	2.3 ± 0.1
Quercetin glycosides^d^	1.6 ± 0.0
Kaempferol glycosides^d^	0.5 ± 0.0
Isorhamnetin glycosides^d^	0.2 ± 0.0

HPLC–DAD, high-performance liquid chromatography with a diode array detector

^a^Data are presented as mean ± SD (*n* = 2)

^b^The content of the substance calculated on cyanidin-3-rutinoside

^c^The content of the substance calculated on quercetin

^d^The content of glycosides calculated on quercetin-3-rutinoside

### Animal study

The use of animals was conducted in compliance with European guidelines for the care, and use of laboratory animals and the animal protocol employed in this study was approved by the local Institutional Animal Care and Use Committee (Olsztyn, Poland). The assessment was conducted on 20 male New Zealand white rabbits aged 34 days and weighing 631 ± 26 g, randomly assigned to one of four groups of five animals each. The rabbits were individually housed in wire net flat-deck cages under standard conditions: temperature was maintained between 19 and 22 °C, relative air humidity was maintained at 60–75 %, the rooms were intensively ventilated, and the photoperiod was regulated (16-h lighting and 8-h darkness). Pelleted diet and tap water were freely available. The detailed composition of the isonitrogenous diets is noted in Table 2. For 4 weeks, the rabbits were subjected to following dietary treatments: control groups C and CF were fed a standard and a high-fat diet, respectively; experimental groups E and EF were fed a standard and a high-fat diet supplemented with 1.5 % blackcurrant polyphenolic extract by weight of the diet (1 % of pure polyphenols). The energy distribution of the standard diet was 21 % from protein, 7 % from fat and 72 % from carbohydrates; the energy from the high-fat diet (which contained 10 % lard) was 17 % from protein, 32 % from fat and 51 % from carbohydrates.

**Table 2:** Composition of the group-specific diets (%)

	Group			
	C	CF	E	EF
Oat	14.00	14.00	14.00	14.00
Wheat bran	10.00	10.00	10.00	10.00
Sunflower meal	16.80	16.80	16.80	16.80
Dried sugar-beet pulp	5.00	5.00	5.00	5.00
Grass meal	16.00	1.00	16.00	1.00
Barley	17.00	19.00	15.00	16.20
Soybean meal	8.20	11.40	8.70	12.50
Cellulose (Vitacel)	10.00	10.00	10.00	10.00
Blackcurrant extract	0.00	0.00	1.50	1.50
Lard	0.00	10.00	0.00	10.00
Additives^a^	3.00	3.00	3.00	3.00
Calculated chemical composition^b^				
Crude protein, %	17.46	17.47	17.45	17.46
Crude fibre, %	14.40	12.70	14.30	12.60
Crude fat, %	2.35	13.50	2.40	13.60
Crude ash, %	4.80	4.70	4.94	4.77
Polyphenols^c^, %	0.00	0.00	1.00	1.00
Metabolisable energy (MJ/kg)	10.12	12.25	10.11	12.24

^a^Additives (per kg of feed): dl-methionine, 2 g; l-lysine, 2 g; monocalcium phosphate 22.6 %, 11 g; salt, 3 g; vitamin–mineral premix (5 g/kg) providing the following nutrients per kg feed: vitamin A, 10,000 IU; vitamin D, 1,800 IU, vitamin E, 15 mg; vitamin K, 4.5 mg; vitamin B1, 0.5 mg; vitamin B2, 4 mg; vitamin B12, 0.01 mg; folic acid, 0.1 mg; pantothenic acid, 7 mg; nicotinic acid, 20 mg; I, 1 mg; Mn, 60 mg; Cu, 5.5 mg; Zn, 75 mg; Fe, 40 mg; Co, 0.3 mg; Se, 0.08 mg

^b^On dry matter basis

^c^Polyphenols originated from the blackcurrant extract

### Collection and analysis of biological material

At the end of the experiment, the rabbits were weighed, anesthetised with sodium pentobarbitone and euthanized by cervical dislocation according to recommendations for experimental animals. Blood samples were taken from the jugular vein into test tubes, and the serum was prepared by solidification and low-speed centrifugation (350×*g*, 10 min, 4 °C). After laparotomy, the selected tissues (liver, kidneys and heart) as well as segments of the gastrointestinal tract (small intestine, caecum and colon, including the content) were removed from each rabbit.

As soon as possible after euthanasia (approximately 10 min), small intestinal, caecal and colonic pH were directly measured using a microelectrode and pH/ION meter (model 301, Hanna Instruments, Vila do Conde, Portugal). The small intestine was weighed with the contents. Samples of ileal (from the central ileum), caecal and colonic contents were immediately transferred to microfuge tubes, which were stored at −70 °C. The segments of the gastrointestinal tract (caecum and colon) were flushed clean with ice-cold saline, blotted on filter paper and, finally, weighed. The dry matter of the small intestinal and caecal digesta was determined at 105 °C for 4 h. In fresh caecal digesta samples, ammonia was extracted and trapped in a solution of boric acid in Conway dishes and was determined according to reported protocols [14]. The total contents of the small intestine were collected, mixed in a vortex and centrifuged at 10,000*g* for 10 min. The supernatant fraction (0.5 mL) was placed in a Brookfield LVDV-II + cone-plate rotational viscometer (CP40; Brookfield Engineering Laboratories Inc., Stoughton, MA, USA), and the viscosity of all samples was measured at a fixed temperature of 37 °C and a shear rate of 60 s^−1^. The activity of the bacterial enzymes (α- and β-glucosidase, α- and β-galactosidase and β-glucuronidase) released into the caecal environment was measured by the rate of *p*- or *o*-nitrophenol released from their nitrophenylglucosides, according to previously described methods [15]. The enzymatic activity was expressed as μm product formed per hour per g of digesta. Caecal and colonic digesta samples were subjected to SCFA analysis, using GC (Shimadzu GC-2010, Kyoto, Japan). The samples (0.2 g) were mixed with 0.2 of formic acid diluted with deionised water and centrifuged at 7,211*g* for 10 min. The supernatant was loaded onto a capillary column (SGE BP21, 30 m × 0.53 mm) using an on-column injector. The initial oven temperature of 85 °C was raised to 180 °C by 8 °C/min and held there for 3 min. The temperatures of the flame ionisation detector and the injection port were 180 and 85 °C, respectively. The sample volume for the GC analysis was 1 μL. The caecal putrefactive SCFA (PSCFA) concentration was calculated as the sum of isobutyrate, isovalerate and valerate concentration in the caecal digesta.

Lipid peroxidation products in the serum and tissue of the internal organs were assessed by reaction with thiobarbituric acid as TBARS, following Uchiyama and Michara [16]. TBARS were determined spectrophotometrically using malondialdehyde to establish the standard curve. The results were expressed as nanomoles of malondialdehyde per gram of tissue or mL of serum. The serum samples were assessed for concentrations of glucose (GL) and lipids, including total (TC), HDL cholesterol and triglycerides (TG) using direct measurement assays (glucose OXY DST, total cholesterol DST, HDL-cholesterol DST and triglycerides DST; Alpha Diagnostic Ltd., Warsaw, Poland). The atherogenic index (AI) of a diet was calculated for each animal according to the formula AI = log (TG/HDL). Another atherogenic index AII was calculated and expressed as AII = (TC-HDL)/HDL. The amount of FFA present in serum of the rabbits was assessed through a coupled reaction to measure non-esterified fatty acid employing a commercially available detection kit (Serum/Plasma Non-Esterified Fatty Acids Detection Kit; Zen-Bio, Research Triangle Park, NC, USA). The concentration of FFA was determined from the optical density observed at 540 nm. To measure insulin concentration, a validated rabbit insulin ELISA kit was employed (Dog, Human and Rabbit Insulin Elisa Kit; Kamiya Biomedical Company, Seattle, USA). The quantity of free insulin present in each serum sample was determined spectrophotometrically at 492 nm. Homoeostasis model assessment for insulin resistance (HOMA-IR) and pancreatic insulin secretion (HOMA-β) were calculated according to the following formulas: HOMA-IR = [fasting insulin (mU/l) × fasting glucose (mmol/l)/22.5], while HOMA-β = [fasting insulin (mU/l) × 20/fasting glucose (mmol/l) − 3.5]. A QUICKI index was calculated with the formula QUICKI = 1/[log fasting insulin (μU/mL)] + [log fasting glucose (mg/dL)]. The total antioxidant status (TAS) of the serum was measured using two-reagent assay (TAS Kit; Randox Laboratories Ltd., Crumlin, United Kingdom). The serum antiradical capacity of water-soluble (ACW) and the antiradical capacity of lipid-soluble (ACL) substances (ACW-Kit and ACL-Kit; Analytik Jena AG, Jena, Germany) were determined by photochemiluminescence detection using a Photochem analyser (Analytik Jena AG, Jena, Germany). In the photochemiluminescence assay, the generation of free radicals was partially eliminated by the reaction with the antioxidants present in the serum samples, and the remaining radicals were quantified by luminescence generation. Ascorbate and Trolox calibration curves were employed to evaluate ACW and ACL, respectively, and the results were expressed as mmol ascorbate or Trolox equivalent/mL serum.

### Statistical analysis

The data are expressed as the means and the pooled standard error of the mean (SEM). The STATISTICA software, version 10.0 (StatSoft Corp., Krakow, Poland), was utilised to determine whether the variables differed among treatment groups. The effects of supplemental dietary blackcurrant extract (E), the type of diet (standard diet or high-fat diet, D) and the interaction between these investigated factors (E × D) were assessed by two-way ANOVA. When the ANOVA identified significant treatment effects, the means were evaluated using Duncan’s multiple range test. The data were checked for normality before the statistical analysis was performed. Differences with *P* < 0.05 were considered statistically significant.

## Results

The blackcurrant extract was characterised by high concentrations of anthocyanins (48.9 %; mainly delphinidin-3-rutinoside, delphinidin-3-glucoside, cyanidin-3-rutinoside) and other polyphenolic compounds, i.e. flavonol aglycones and glycosides (Table 1). The initial body weight of the rabbits did not differ among groups (data not shown). The addition of blackcurrant extract had no effect (*P* > 0.05) on final body weight, body weight gain or food intake during the study (Table 3) . The growth rate and food intake were significantly affected by the type of diet; high-fat diets increased the BWG and decreased the food intake of the rabbits. The small intestinal digesta of the animals fed with additional lard was characterised by an increased (*P* < 0.05) viscosity rate as well as a significant decrease in pH. The dietary addition of polyphenolic extract was also associated with a decrease in small intestinal pH.

**Table 3:** Final body weight (BW, kg), body weight gain (BWG, g/day), food intake mass (FI, g/day) and basic intestinal indices in rabbits fed experimental diets

	BW	BWG	FI	Small intestine				Caecum				
				Weight^a^	DM^b^	DV^c^	pH	Tissue^d^	Digesta^d^	NH_3_, mg/g	DM^b^	pH
Group												
C	1.78	41.0	117	30.1	10.0	1.41	7.61	14.4	41.7	0.305	21.8	6.62
CF	2.28	58.9	94.4	36.7	12.8	1.57	7.53	12.4	33.5	0.507	21.0	6.71
E	1.93	46.6	116	34.5	9.16	1.42	7.50	16.0	44.1	0.257	21.5	6.45
EF	2.21	56.6	96.0	34.4	12.1	1.52	7.16	13.7	37.6	0.404	20.5	6.59
SEM	0.071	2.557	3.756	1.076	0.649	0.026	0.040	0.399	1.439	0.026	0.331	0.060
Extract (E)												
Without	2.03	50.0	106	33.4	11.4	1.49	7.57	13.4	37.6	0.406	21.4	6.66
With	2.07	51.6	106	34.5	10.3	1.47	7.33	14.8	40.9	0.331	21.0	6.52
*P value*	*0.707*	*0.701*	*0.987*	*0.614*	*0.372*	*0.641*	*0.026*	*0.026*	*0.194*	*0.027*	*0.559*	*0.073*
Diet (D)												
Standard	1.85	43.8	116	32.3	9.60	1.41	7.55	15.2	42.9	0.281	0.217	6.54
With fat	2.25	57.8	95.2	35.5	12.1	1.54	7.34	13.0	35.6	0.456	0.208	6.65
*P value*	*0.004*	*0.004*	*0.004*	*0.131*	*0.060*	*0.013*	*0.047*	*0.002*	*0.007*	*0.000*	*0.208*	*0.156*
Interaction E × D												
*P value*	*0.345*	*0.364*	*0.813*	*0.123*	*0.843*	*0.526*	*0.212*	*0.773*	*0.715*	*0.387*	*0.874*	*0.770*

Groups C and CF were fed standard and high-fat diets, respectively; E and EF were fed standard and high-fat diets with 1.5 % blackcurrant polyphenolic extract, respectively (*n* = 5)

^a^Full weight with contents, g/kg BW; ^b^ Dry matter of digesta,  %; ^c^ Digesta viscosity, mPa s; ^d ^g/kg BW

**Fig. 1:**
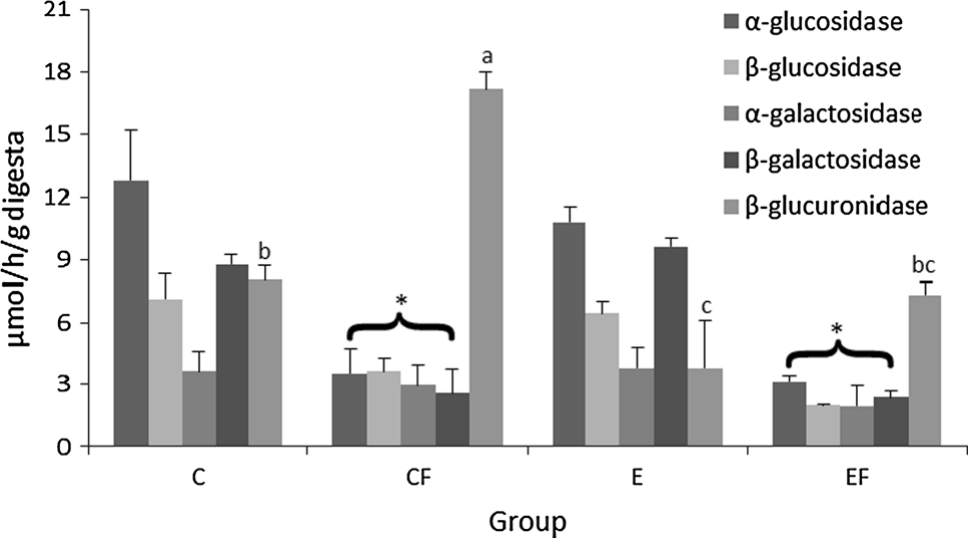
Microbial enzyme activities in the caecal digesta of rabbits fed experimental diets. Groups C and CF were fed standard and high-fat diets, respectively; E and EF were fed standard and high-fat diets with 1.5 % blackcurrant polyphenolic extract, respectively (*n* = 5). Mean values with unlike letters (*a*, *b*, *c*) are significantly different at *P* < 0.05; differences among groups *C*, *CF*, *E* and *EF* are indicated with letters only in the case of a statistically significant interaction (Extract × Diet, *P* < 0.05). *Mean values are different between high-fat diets and standard diets (*P* < 0.01)

Increased dietary lard significantly reduced (*P* < 0.05) the relative mass of the caecal tissue and its contents and increased the caecal concentration of ammonia compared with the standard diet (Table 3). The supplemental blackcurrant extract significantly increased relative caecal tissue mass and caused a significant decrease in caecal ammonia concentration (*P* < 0.05 vs. no extract supplementation). The high-fat feeding regimen significantly suppressed the activity of bacterial glycolytic enzymes, α- and β-glucosidases as well as α- and β-galactosidases, released into the caecal environment (Fig. 1). A significant interaction effect (*P* < 0.05) between diet type and supplementation was observed on caecal β-glucuronidase activity. The high-fat diet without blackcurrant extract resulted in the highest β-glucuronidase activity compared with all other groups, but the addition of the extract to the high-fat diet significantly decreased β-glucuronidase activity to the level observed in the C group. The lowest activity of this bacterial enzyme in the caecal digesta was noted in the E rabbits (*P* < 0.05 vs. C and CF groups, Fig. 1). Irrespective of the diet type, dietary supplementation with blackcurrant extract significantly decreased the caecal concentration of putrefactive SCFA (Table 4). The dietary blackcurrant extract, when added to a standard diet, caused a significant decrease in caecal butyric acid concentration; such an effect was not observed when the extract was added to the high-fat diet (see the significant interaction E × D). The rabbits’ total caecal concentration of SCFA was affected neither by the addition of extract nor the type of diet. When the colonic digesta was analysed, the concentration of PSCFA was significantly reduced by treatment with blackcurrant extract while the high-fat diet tended to cause the opposite effect. The dietary lard significantly increased colonic concentration of total SCFA and, surprisingly, raised colonic digesta pH.

**Table 4:** Short-chain fatty acid (SCFA) concentration (μmol/g digesta) in the gut digesta of rabbits

	Caecal digesta					Colonic digesta		
	C2	C3	C4	PSCFA	Total SCFA	PSCFA	Total SCFA	pH
Group								
C	19.1	2.99	1.71^a^	0.947	24.8	0.598	16.0	6.66
CF	18.1	3.09	1.79^a^	0.761	23.8	0.762	17.8	6.88
E	18.8	2.79	1.10^b^	0.494	23.2	0.299	15.1	6.52
EF	20.5	2.90	2.04^a^	0.634	25.9	0.349	18.5	6.88
SEM	0.717	0.142	0.110	0.055	0.770	0.050	0.582	0.068
Extract (E)								
Without	18.6	3.04	1.75	0.854	24.3	0.676	16.9	6.77
With	19.6	2.85	1.57	0.501	24.5	0.324	16.8	6.70
*P value*	*0.523*	*0.532*	*0.298*	*0.000*	*0.875*	*0.000*	*0.957*	*0.610*
Diet (D)								
Standard	19.0	2.89	1.40	0.720	24.0	0.444	15.6	6.59
With fat	19.3	2.99	1.92	0.634	24.9	0.556	18.2	6.88
*P value*	*0.820*	*0.746*	*0.007*	*0.269*	*0.591*	*0.055*	*0.027*	*0.042*
Interaction E × D								
*P value*	*0.385*	*0.992*	*0.020*	*0.205*	*0.257*	*0.272*	*0.485*	*0.610*

Groups C and CF were fed standard and high-fat diets, respectively; E and EF were fed standard and high-fat diets with 1.5 % blackcurrant polyphenolic extract, respectively (*n* = 5)

*PSCFA* putrefactive SCFA (sum of isobutyric, isovaleric and valeric acids)

^a,b^Mean values within a column with unlike superscript letters are significantly different (*P* < 0.05); differences among groups C, CF, E and EF are indicated with superscripts only in the case of a statistically significant interaction E × D (*P* < 0.05)

Irrespective of blackcurrant extract supplementation, the lard-supplemented diet caused a significant increase in relative liver mass compared with the livers of rabbits fed standard diets (Table 5). TBARS concentration in all analysed organs (liver, kidney and heart) was significantly higher in animals fed a high-fat diet. In this respect, the dietary supplementation with blackcurrant extract tended to decrease the TBARS concentration in the kidney tissue (*P* = 0.088 vs. no supplementation). For the serum concentration of TBARS, the two-way ANOVA indicated a significant interaction E × D (*P* = 0.026); dietary blackcurrant extract caused a significant decrease in the serum TBARS level of rabbits fed a high-fat diet, but this effect was not observed in animals on a standard diet. Regardless of extract consumption, the addition of 10 % lard caused significant decreases in the levels of serum TAS and ACW and had no effect on the serum ACL level. The addition of blackcurrant extract had the opposite effect on the aforementioned parameters and caused a significant increase in serum TAS and ACW levels and a statistically significant tendency (*P* = 0.068) towards a higher ACL level.

**Table 5:** Indices of antioxidant status in rabbits fed experimental diets

	Liver		Kidneys		Heart		Serum			
	Weight g/kg BW	TBARS nmol/g	Weight g/kg BW	TBARS nmol/g	Weightg/kg BW	TBARS nmol/g	TBARS nmol/mL	TAS mmol/L	ACW mmol/mL	ACL mmol/mL
Group										
C	24.8	77.7	6.36	125	2.58	65.0	63.5^c^	1.11	0.065	0.089
CF	34.3	93.1	6.11	155	2.28	71.1	169^a^	0.884	0.054	0.088
E	25.4	75.2	6.26	120	2.55	63.8	46.6^c^	1.19	0.078	0.095
EF	33.6	89.7	6.33	141	2.39	67.8	118^b^	1.06	0.064	0.093
SEM	1.147	2.008	0.202	3.940	0.072	0.953	11.44	0.029	0.002	0.001
Extract (E)										
Without	29.6	85.4	6.23		2.43	68.0	116	0.998	0.059	0.089
With	29.5	82.5	6.29	131 140	2.47	65.5	82.5	1.12	0.071	0.094
*P value*	*0.968*	*0.186*	*0.896*	*0.088*	*0.775*	*0.164*	*0.000*	*0.000*	*0.000*	*0.068*
Diet (D)										
Standard	25.1	76.5	6.31	122	2.57	64.4	55.1	1.15	0.071	0.092
With fat	34.0	91.4	6.22	148	2.34	69.5	143	0.972	0.059	0.090
*P value*	*0.000*	*0.000*	*0.838*	*0.000*	*0.128*	*0.005*	*0.000*	*0.000*	*0.000*	*0.583*
Interaction E × D										
*P value*	*0.626*	*0.850*	*0.724*	*0.440*	*0.653*	*0.522*	*0.026*	*0.099*	*0.559*	*0.774*

Groups C and CF were fed standard and high-fat diets, respectively; E and EF were fed standard and high-fat diets with 1.5 % blackcurrant polyphenolic extract, respectively (*n* = 5)

*ACW* antioxidant capacity of water-soluble substances in the serum (mmol AA/mL), *ACL* antioxidant capacity of lipid-soluble substances in the serum (mmol Trolox/mL), *TAS* total antioxidant status (mmol/L), *TBARS* thiobarbituric acid-reactive substances

^a,b,c^Mean values within a column with unlike superscript letters are significantly different (*P* < 0.05); differences among groups C, CF, E and EF are indicated with superscripts only in the case of a statistically significant interaction E × D (*P* < 0.05)

For the serum concentrations of TG, TC and non-HDL-C, the two-way ANOVA revealed significant interactions E × D (*P* = 0.011, *P* = 0.012, *P* = 0.007, respectively); dietary blackcurrant extract caused a significant decrease in the serum TG level of rabbits fed a high-fat diet while such an effect was not observed in animals on a standard diet (Table 6). The rabbits from the CF group were characterised by the highest concentrations of serum TC and non-HDL-C fractions (*P* < 0.05 vs. all other groups); supplementation with blackcurrant extract in animals fed a high-fat diet reduced TC and non-HDL-C concentrations to the levels observed in the C and E animals. The treatments had no effect on serum HDL-C concentration but significantly changed the values of the HDL-C/TC profile; the addition of dietary lard significantly reduced that profile value and the dietary blackcurrant extract increased the profile value. The high-fat diets caused a significant increase in serum FFA and insulin concentrations while the blackcurrant extract produced the opposite (insulin levels exhibited a statistical tendency towards lower concentration with supplementation). Compared with a standard diets, the addition of lard led to significant increase in the AI [log(TG/HDL-C], the AII [(TC-HDL-C)/HDL-C], the HOMA-β, the HOMA-IR and the QUICKI (in the latter two cases, a tendency very close to statistical significance was observed, *P* = 0.052 and *P* = 0.051, respectively) values (Table 7). The addition of blackcurrant extract caused a significant decrease in the values of AI and AII indexes regardless of diet type.

**Table 6:** Biochemical indices of the serum in experimental rabbits

	GL (mmol/L)	TG (mmol/L)	TC (mmol/L)	HDL-C (mmol/L)	HDL-C % of TC	Non-HDL-C (mmol/L)	FFA (mmol/L)	Insulin (pmol/L)
Group								
C	6.04	1.05^c^	2.11^b^	1.01	48.0	1.11^b^	394	127
CF	5.81	2.29^a^	3.18^a^	1.19	37.6	1.99^a^	1,243	153
E	6.28	1.06^c^	2.26^b^	1.17	51.6	1.09^b^	279	125
EF	5.88	1.57^b^	2.45^b^	1.15	47.0	1.30^b^	1,059	144
SEM	0.109	0.130	0.118	0.039	1.476	0.097	99.44	0.018
Extract (E)								
Without	5.92	1.67	2.64	1.10	42.8	1.55	818	140
With	6.08	1.32	2.36	1.16	49.3	1.20	669	134
*P value*	*0.483*	*0.014*	*0.080*	*0.424*	*0.003*	*0.005*	*0.031*	*0.093*
Diet (D)								
Standard	6.16	1.05	2.19	1.09	49.8	1.10	336	126
With fat	5.85	1.93	2.81	1.17	42.3	1.65	1,151	149
*P value*	*0.178*	*0.000*	*0.001*	*0.332*	*0.001*	*0.000*	*0.000*	*0.000*
Interaction E × D								
*P value*	*0.712*	*0.011*	*0.012*	*0.202*	*0.141*	*0.007*	*0.594*	*0.240*

Blood samples were taken from overnight food-deprived animals

Groups C and CF were fed standard and high-fat diets, respectively; E and EF were fed standard and high-fat diets with 1.5 % blackcurrant polyphenolic extract, respectively (*n* = 5)

*FFA* free fatty acids, *GL* glucose, *HDL-C* HDL cholesterol, *non-HDL-C* the difference between TC and HDL-C, *TC* total cholesterol, *TG* triglycerides

^a,b,c^Mean values within a column with unlike superscript letters are significantly different (*P* < 0.05); differences among groups C, CF, E and EF are indicated with superscripts only in the case of a statistically significant interaction E × D (*P* < 0.05)

**Table 7:** Indexes of atherogenicity and insulin resistance calculated for rabbits fed experimental diets

	AI	AII	HOMA-IR	HOMA-β	QUICKI
Group					
C	0.020	1.09	4.91	148	0.303
CF	0.283	1.70	5.72	203	0.298
E	−0.054	0.944	5.02	131	0.302
EF	0.133	1.44	5.42	177	0.299
SEM	0.036	0.081	0.151	8.958	0.010
Extract (E)					
Without	0.151	1.40	5.32	175	0.301
With	0.039	1.04	5.22	154	0.301
*P value*	*0.031*	*0.003*	*0.739*	*0.142*	*0.879*
Diet (D)					
Standard	−0.017	1.02	4.97	139	0.303
With fat	0.208	1.42	5.57	190	0.299
*P value*	0.000	0.001	0.052	0.002	0.051
Interaction E × D					
*P value*	*0.435*	*0.063*	*0.478*	*0.754*	*0.490*

AI = log(TG/HDL-C); AII = (TC-HDL-C)/HDL-C; HOMA-IR = [fasting insulin (mU/L) × fasting glucose (mmol/L)/22.5]; ^5^HOMA-β = [fasting insulin (mU/L) × 20/fasting glucose (mmol/L) − 3.5]; QUICKI = 1/[log fasting insulin (μU/mL)] + [log fasting glucose (mg/dL)]

Groups C and CF were fed standard and high-fat diets, respectively; E and EF were fed standard and high-fat diets with 1.5 % blackcurrant polyphenolic extract, respectively (*n* = 5)

## Discussion

In the present study, the dietary content of blackcurrant polyphenols was approximately 1 %. Taking into account the estimated food intake among the different groups, the average daily intake of these bioactive constituents approached or slightly exceeded 1 g per day. This amount is a relatively high dietary level, but it is a level possible to obtain in a common human diet [17]. Scalbert and Williamson [18] suggested that total polyphenol intake most likely exceeds 1 g/d when people eat several servings of fruits and vegetables per day. It is well known that polyphenols that are not absorbed in the stomach or small intestine are carried to the lower bowel. Moreover, polyphenols that are absorbed, metabolised in the liver and excreted in the bile or directly from the enterocyte back to the small intestine also reach the caecal/colonic segments in a different chemical form, e.g. as a glucuronide [18]. In this case, the activity of bacterial β-glucuronidase may help to release polyphenol aglycones in the colonic lumen, but it should be clearly stated that the healthy body employs many different detoxification pathways, in the liver and elsewhere, of which glucuronidation is of paramount importance. Glucuronidation removes several toxic and potentially toxic chemicals from our system, such as polycyclic aromatic hydrocarbons, steroid hormones, some nitrosamines, heterocyclic amines, some fungal toxins and aromatic amines [19]. Therefore, a high level of activity of bacterial β-glucuronidase should be considered a negative result of dietary habits. The results of the present showed that the consumption of blackcurrant extract can beneficially modulate caecal microbiota enzymatic activity, as manifested by β-glucuronidase; the extract decreased this enzyme activity in both standard and high-fat diets by 48.1 and 42.7 %, respectively. Molan et al. [3] in the study on rats gavaged with a commercial supplement containing blackcurrant extract exhibited significant reduction in the caecal β-glucuronidase activity by 31.5 %. The supplementation of polyphenolic extract from blackcurrant fruit also triggered a desirable reduction in caecal ammonia level compared with the dietary treatment without the extract. The balance between the ammonia produced and absorbed by bacteria and intestinal cells is vital for the ammonia concentration, which may change the metabolism and morphology of the intestinal epithelium and, thus, leading to carcinogenesis [20]. The production of ammonia is closely related to bacterial breakdown of urea and undigested protein substances; other putrefactive catabolites associated with the microbiotic metabolism of nitrogenous compounds that enter the lower part of the GIT are putrefactive short-chain fatty acids (PSCFA), which may also point to poorer dietary protein utilisation. Dietary supplementation with blackcurrant extract was characterised by the lowest individual (data not shown) and total PSCFA levels. Note that several putrefactive compounds produced during fermentation in the large gut (e.g. ammonia, PSCFA) are partly responsible for the malodour of digesta [21]. Such a beneficial decrease in putrefaction in the distal intestine resulting from the consumption of a high level of polyphenol-rich blackcurrant extract is an important novelty found in the present study. However, large differences in digestive physiology between rabbits and humans have to be kept in mind when relating the results to human health.

In the present study, supplementation with blackcurrant extract did not reduce the level of diet-induced obesity. Prior et al. [22] observe a significant reduction in the obesity rates of mice following supplementation with berry anthocyanins, but not whole berries, despite the similar dietary levels of polyphenols of both treatments. This observation supports the thesis that, in the search for effective food supplements to reduce the risk of disturbances induced by an inappropriate diet, polyphenolic products have to be seriously considered not just because such products are very convenient to reach the desired daily intake. As expected in the present study, the increased dietary intake of lard led to negative changes in the antioxidant status of the rabbits’ bodies as manifested by increased TBARS concentration in several tissues (liver, kidney, heart and blood serum). As the oxidation products of lipids, TBARS illustrate the oxidative state of lipids localised primarily in cell membranes and thus serve as a biomarker characterising the systemic pro-/antioxidant equilibrium. It has been postulated that polyphenols are potent scavengers of reactive forms of oxygen and nitrogen and are able to inhibit the activity of enzymes and chelate metal ions that catalyse oxidation reactions [23]. In the current study, blackcurrant extract inhibited lipid peroxidation induced by high dietary fat as evidenced by lower concentrations of TBARS in the kidneys and serum in the rabbits treated with the extract. The suppression of lipid peroxidation observed in the kidneys of rabbits fed diets enriched with the extract was likely the effect of polyphenolic compounds that may increase filtration in these organs and inactivate free radicals. The suppression of lipid peroxidation by blackcurrant has also been demonstrated in in vivo experiments in which the administration of blackcurrant juice substantially lowered the level of TBARS in the plasma and in the liver of rats [24, 25]. Other indices measured in our study, i.e. higher serum levels of TAS, ACW and ACL, support the hypothesis that blackcurrant extract favourably affected final antioxidant status not only related to lipids and lipid-soluble substances circulating in blood. Manach et al. [17] highlight the fact that the hydrophobicity of polyphenols is between that of vitamin C (highly hydrophilic) and that of vitamin E (highly hydrophobic). As demonstrated in the present study, compounds of blackcurrant extract are expected to act beneficially at water–lipid interfaces.

Hyperlipidaemia and increased FFA turnover are the primary alterations of lipid metabolism observed in obesity. This reflects increased lipolysis in conjunction with insufficient plasma clearance leading to fat accumulation. It is noteworthy that rabbits consuming a high-fat diet ad libitum experience haemodynamic, neurohumoral and increased lipid turnover changes similar to those observed in obese humans [26]. Interestingly, some authors report favourable systemic reaction in the blood lipid profile of patients following long-term intake of berries, including blackcurrants [27]. Qin et al. [28] also found that an anthocyanin extract from bilberry and blackcurrant significantly increased HDL-C concentration in dyslipidaemic patients’ blood. In the present experiment, dietary treatment with blackcurrant extract did not affect HDL-C concentration in rabbits’ serum but effectively reduced TG and TC levels; the latter levels were reduced via a substantial decrease in the non-HDL fraction. These beneficial changes in animals fed diets supplemented with the extract were supported by a decreased HDL-C/TC profile as well as by a substantial decrease in the values of calculated atherogenic indexes, i.e. the AI expressed as log(TG/HDL-C) and the AII expressed as (TC-HDL-C)/HDL-C. A number of in vivo studies have already shown that berry extracts rich in polyphenols, including blackcurrant extracts, have lipid-lowering activities. However, this is the first time that a by-product of blackcurrant processing ingested in relatively high amounts can distinctly attenuate hyperlipidaemia. Different mechanisms may be involved in the hypolipidaemic action of berry phenolic compounds. Recent studies have demonstrated that bioactive constituents from different berry fruits are potent inhibitors of pancreatic lipase [29], an enzyme involved in the digestion and absorption of dietary fat. Other studies [30] have pointed at flavonoids as potent inhibitors of two key enzymes involved in the extracellular and intracellular cholesterol metabolism, i.e. 3-hydroxy-3-methyl-glutaryl-CoA reductase and acyl CoA:cholesterol *O*-acyltransferase. A study on Watanabe heritable hyperlipidemic rabbits conducted by Finne Nielsen et al. [31] provided interesting, but to some extent worrying, data regarding the adverse effects of pure anthocyanins isolated from blackcurrants, but not blackcurrant juice, on plasma LDL-cholesterol levels. These results have suggested that other bioactive components of blackcurrants than anthocyanins are responsible for the reduction in atherosclerosis symptoms. In the present study, compounds of blackcurrant extract exerted some beneficial, but less defined, effects on the indices of glucose metabolism. The values for HOMA-β, HOMA-IR and QUICKI indexes did not differ significantly between groups treated without and with blackcurrant extract. However, the significantly lower level of FFA and a statistical tendency towards lower serum concentrations of insulin in rabbits fed diets with blackcurrant extract suggest the potential utility of blackcurrant bioactive compounds in the control of insulinaemia. The available literature clearly suggests that increased circulating FFA may directly contribute to the underlying pathophysiology of type 2 diabetes, particularly, through the development of insulin resistance both in the periphery and the liver [32]. Polyphenolic compounds, especially anthocyanins, seem to influence the cascade of reactions associated with insulin secretion and signalling [33], especially modulating adipokines, increasing the expression of GLUT4, decreasing the expression of retinol binding protein in blood, activating AMP-activated protein kinase and reducing oxidative stress [34].

## Conclusion

The consumption of a polyphenol-rich extract from blackcurrant pomace at an average amount of 1.5 g per day for 4 weeks by rabbits fed a high-fat diet produces beneficial modification of the serum lipids and the antioxidant status of the body as well measurements of large-bowel function. The possibility of ameliorating insulin resistance is unclear. A by-product of blackcurrant processing ingested in relatively high amounts may be also a useful therapeutic option in ameliorating dysfunctions related to obesity and its complications.

## References

[1] Rosenblat M, Volkova N, Attias J, Mahamid R, Aviram M (2010). Consumption of polyphenolic-rich beverages (mostly pomegranate and black currant juices) by healthy subjects for a short term increased serum antioxidant status, and the serum’s ability to attenuate macrophage cholesterol accumulation. Food Funct.

[2] Bishayee A, Mbimba T, Thoppil RJ, Haznagy-Radnai E, Sipos P, Darveh AS, Folkesson HG, Hohmann J (2011). Anthocyanin-rich blackcurrant (*Ribes nigrum* L.) extract affords chemoprevention against diethylnitrosamine-induced hepatocellular carcinogenesis in rats. J Nutr Biochem.

[3] Molan AL, Liu Z, Kruger M (2010). The ability of blackcurrant extracts to positively modulate key markers of gastrointestinal function in rats. World J Microbiol Biotechnol.

[4] Sojka M, Guyot S, Kolodziejczyk K, Krol B, Baron A (2009) Composition and properties of purified phenolics preparation obtained from an extract of industrial blackcurrant (*Ribes nigrum* L) pomace. J Hortic Sci Biotech ISAFRUT Sp Iss 100–106

[5] Werlein H-D, Kutemeyer C, Schatton G, Hubbermann EM, Schwarz K (2005). Influence of elderberry and blackcurrant concentrates on the growth of microorganisms. Food Control.

[6] Spencer JP (2003). Metabolism of tea flavonoids in the gastrointestinal tract. J Nutr.

[7] Juskiewicz J, Zdunczyk Z, Jankowski J, Krol B, Milala J (2008). Gastrointestinal tract metabolism of young turkeys fed diets supplemented with pure nystose or a fructooligosaccharide mixtue. Arch Anim Nutr.

[8] Frejnagel S, Juśkiewicz J (2011). Dose-dependent effects of polyphenolic extracts from green tea, blue-berried honeysuckle, and chockeberry on rat caecal fermentation processes. Planta Med.

[9] Puupponen-Pimiä R, Nohynek L, Hartmann-Schmidlin S, Kähkönen M, Heinonen M, Määttä-Riihinen K, Oksman-Caldentey KM (2005). Berry phenolics selectively inhibit the growth of intestinal pathogens. J Appl Microbiol.

[10] Delzenne NM, Cani PD (2011). Interaction between obesity and the gut microbiota: relevance in nutrition. Ann Rev Nutr.

[11] de Wit N, Derrien M, Bosch-Vermeulen H, Oosterink E, Keshtkar S, Duval C, de Vogel-van den Bosch J, Kleerebezem M, Muller M, van der Meer R (2012). Saturated fat stimulates obesity and hepatic steatosis and affects gut microbiota composition by an enhanced overflow of dietary fat to the distal intestine. Am J Physiol Gastrointest Liver Physiol.

[12] AOAC (2005). Official methods of analysis of the association of the official analytical chemists.

[13] Sojka M, Krol B (2009). Composition of industrial seedless black currant pomace. Eur Food Res Technol.

[14] Jurgonski A, Juskiewicz J, Zdunczyk Z (2008). Comparative effects of different dietary levels of cellulose and fructooligosaccharides on fermentative processes in the caecum of rats. J Anim Feed Sci.

[15] Juskiewicz J, Zdunczyk Z, Zary-Sikorska E, Krol B, Milala J, Jurgonski A (2011). Effect of the dietary polyphenolic fraction of chicory root, peel, seed and leaf extracts on caecal fermentation and blood parameters in rats fed diets containing prebiotic fructans. Br J Nutr.

[16] Uchiyama M, Mihara M (1978). Determination of malonaldehyde precursor in tissues by thiobarbituric acid test. Anal Biochem.

[17] Manach C, Scalbert A, Morand C, Remesy C, Jimenez L (2004). Polyphenols: food sources and bioavailability. Am J Clin Nutr.

[18] Scalbert A, Williamson G (2000). Dietary intake and bioavailability of polyphenols. J Nutr.

[19] Zheng Z, Fang JL, Lazarus P (2002). Glucuronidation: an important mechanism for detoxification of benzo[a]pyrene metabolites in aerodigestive tract tissues. Drug Metab Dispos.

[20] Juskiewicz J, Zdunczyk Z, Frejnagel S (2007). Caecal parameters of rats fed diets supplemented with inulin in exchange for sucrose. Arch Anim Nutr.

[21] Swanson KS, Grieshop CM, Flickinger EA, Bauer LL, Wolf BW, Chow J, Garleb KA, Williams JA (2002). Fructooligosaccharides and *Lactobacillus acidophilus* modify bowel function and protein catabolites excreted by healthy humans. J Nutr.

[22] Prior RL, Wu X, Gu L, Hager TJ, Hager A, Howard LR (2008). Whole berries versus berry anthocyanins: interactions with dietary fat levels in the C57BL/6 J mouse model of obesity. J Agric Food Chem.

[23] Willcox JB, Curb JD, Rodriguez BL (2008). Antioxidants in cardiovascular health and disease: key lessons from epidemiologic studies. Am J Cardiol.

[24] Breinholt VM, Nielsen SE, Knuthsen P, Lauridsen ST, Daneshvar B, Sorensen A (2003). Effects of commonly consumed fruit juices and carbohydrates on redox status and anticancer biomarkers in female rats. Nutr Cancer.

[25] Farombi EO, Hansen M, Ravn-Haren G, Moller P, Dragsted LO (2004). Commonly consumed and naturally occurring dietary substances affect biomarkers of oxidative stress and DNA damage in healthy rats. Food Chem Toxicol.

[26] Zhang X-J, Chinkes DL, Aarsland A, Herndon DN (2008). Lipid metabolism in diet-induced obese rabbits is similar to that of obese humans. J Nutr.

[27] Erlund I, Koli R, Alfthan G, Marniemi J, Puukka P, Mustonen P, Mattila P, Jula A (2008). Favorable effects of berry consumption on platelet function, blood pressure, and HDL cholesterol. Am J Clin Nutr.

[28] Qin Y, Xia M, Ma J, Hao JT, Liu J, Mou HY, Cao L, Ling WH (2009). Anthocyanin supplementation improves serum LDL- and HDL-cholesterol concentrations associated with the inhibition of cholesteryl ester transfer protein in dyslipidemic subjects. Am J Clin Nutr.

[29] McDougall GJ, Kulkarnia NN, Berry DS (2009). Polyphenols inhibit pancreatic lipase activity in vitro. Food Chem.

[30] Bok S-H, Lee S-H, Park Y-B, Bae K-H, Son K-H, Jeong T-S, Choi M-S (1999). Plasma and hepatic cholesterol and hepatic activities of 3-hydroxy-3-methyl-glutaryl-CoA reductase and acyl CoA: cholesterol transferase are lower in rats fed citrus peel extract or a mixture of citrus bioflavonoids. J Nutr.

[31] Finne Nielsen IL, Elbol Rasmussen S, Mortensen A, Ravn-Haren G, Ma HP, Knuthsen P, Fischer Hansen B, McPhail D, Freese R, Breinholt V, Frandsen H, Dragsted LO (2005). Anthocyanins increase low-density lipoprotein and plasma cholesterol and do not reduce atherosclerosis in Watanabe Heritable Hyperlipidemic rabbits. Mol Nutr Food Res.

[32] Boden G, Shulman GI (2002). Free fatty acids in obesity and type 2 diabetes: defining their role in the development of insulin resistance and β-cell dysfunction. Eur J Clin Invest.

[33] Jayaprakasam B, Vareed SK, Olson LK, Nair MG (2005). Insulin secretion by bioactive anthocyanins and anthocyanidins present in fruits. J Agric Food Chem.

[34] Soriano Sancho RA, Pastore GM (2012). Evaluation of the effects of anthocyanins in type 2 diabetes. Food Res Int.

